# 基因富集及*meta*分析筛选非小细胞肺癌发生发展关键基因的研究

**DOI:** 10.3779/j.issn.1009-3419.2012.07.05

**Published:** 2012-07-20

**Authors:** 文武 何, 磊 冼, 永勇 王, 艳玲 胡, 铭伍 陈

**Affiliations:** 530021 南宁，广西医科大学第一附属医院心胸外科 Department of Cardiothoracic Surgery, the First Affiliated Hospital, Guangxi Medical University, Nanning 530021, China

**Keywords:** 肺肿瘤, 基因富集, *meta*分析, 通路, 关键基因, Lung neoplasms, Gene set-enrichment analysis, *Meta*-analysis, Pathway, Key gene

## Abstract

**背景与目的:**

非小细胞肺癌（non-small cell lung cancer, NSCLC）是全球最常见的恶性肿瘤之一，其发病遗传机制仍不清楚。本研究旨在筛选影响NSCLC发生发展的关键基因和通路，为NSCLC发病遗传机制及靶向治疗的研究奠定科学基础。

**方法:**

运用基因组富集分析（gene set enrichment analysis, GSEA）以及对单套数据集单个基因元分析（*meta*-analysis, *meta*）的方法，筛选出在转录水平上影响NSCLC发生发展的关键通路和基因。

**结果:**

通过GSEA和*meta*两种分析方法得出的通路中，重叠性较高的主要为粘着斑通路和细胞骨架肌动蛋白调控通路。在粘着斑通路中31个基因具有统计学意义（*P* < 0.05）；细胞骨架肌动蛋白调控通路中32个基因具有统计学意义（*P* < 0.05）。

**结论:**

粘着斑通路和细胞骨架肌动蛋白调控通路可能与NSCLC的发生发展有重要的联系，后续本研究小组将对这两条通路中的具有统计学意义的基因进行生物功能学上的验证。

## Abstract (en)

**Background and objective:**

Non-small cell lung cancer (NSCLC) is one of the most common malignant tumors; however, its causes are still not completely understood. This study was designed to screen the key genes and pathways related to NSCLC ℃currence and development and to establish the scientific foundation for the genetic mechanisms and targeted therapy of NSCLC.

**Methods:**

Both gene set-enrichment analysis (GSEA) and *meta*-analysis (*meta*) were used to screen the critical pathways and genes that might be corretacted with the development and progression of lung cancer at the transcription level.

**Results:**

Using the GSEA and *meta* methods, focal adhesion and regulation of actin cytoskeleton were determined to be the more prominent overlapping significant pathways. In the focal adhesion pathway, 31 genes were statistically significant (*P* < 0.05), whereas in the regulation of actin cytoskeleton pathway, 32 genes were statistically significant (*P* < 0.05).

**Conclusions:**

The focal adhesion and the regulation of actin cytoskeleton pathways might play important roles in the ℃currence and development of NSCLC. Further studies are needed to determine the biological function for the positiue genes.

非小细胞肺癌（non-small cell lung cancer, NSCLC）是全球最常见恶性肿瘤之一，2011年最新在线出版的全球癌症统计^[1]^表明，肺癌是男性中最常见的癌症，也是男性死亡最常见的病因；在女性常见的癌症中，排在第4位，也是第2位死亡病因。随着肿瘤发病机制及其生物学行为研究的不断深入，越来越多的焦点聚向了以特异性高、不良反应轻为特点的分子靶向治疗。在肿瘤发生发展过程中有大量疾病关键基因和伴随基因参与癌基因的扩增过程，但是如何将影响肿瘤发生发展的关键性分子改变从伴随性改变中识别出来是目前肿瘤研究领域的重要挑战之一。近年来随着人类基因组测序工程的完成，研究者开始把基因芯片广泛应用于肿瘤学研究，通过改变实验条件或实验标本对全基因组mRNA的表达使用基因芯片进行检测，得到了无数基因芯片数据，继而产生了基因芯片数据库（gene expression omnibus, GEO）^[2]^。但是如何挖掘出与疾病发生发展密切相关的关键基因作为治疗疾病的靶点，仍然是一个巨大的挑战。为了能解决这个问题，Subramanian等^[3]^提出基因组富集（gene set enrichment analysis, GSEA）分析，该方法能在病例对照类型数据中基于基因组系统水平挖掘影响疾病发生发展的关键基因通路。通过分析一组处于2种生物学状态的基因表达谱芯片数据，了解他们在特定功能基因集中的表达状况以及这种表达状况是否存在某种统计学意义。另外，因为实验平台、样本、标化方法、分析方法等问题的存在，不同实验室的芯片数据有很多的差异，元分析（*meta*-analysis, *meta*）是一种可行的解决方法，可对同一个问题所发表相关研究报告的结果进行收集、统计上的整合，以期获得更准确或更多的结果^[4]^。考虑通过探索人类NSCLC形成过程中共同拥有的基因改变，可能筛选出影响NSCLC发生发展的关键基因，因此本研究应用GSEA和*meta*分析方法对标准化以后的3套NSCLC全基因组表达芯片数据进行分析，期望筛选出可能影响NSCLC发生发展的关键通路和基因，为NSCLC发病机制的研究提供重要的理论基础。

## 1 材料与方法

### 1.1 材料

本研究设定搜索关键词为“non-small cell lung cancer”，限制研究类型为“expression profiling by array”，在GEO数据库（http://www.ncbi.nlm.gov/geo/）中搜索，结果提供与NSCLC有关的全基因组表达芯片数据有114套。制定数据集的纳入标准为：①数据集必须是有文献支持的全基因组mRNA表达芯片数据；②每套数据均有NSCLC癌组织和正常组织对照；③本次均考虑原始或者经标准化数据集；④每套数据集必须包括3个以上样本；⑤数据集采用的样本必须是人体肺组织。最后，只有3套样本数据集纳入研究（表 1）。

**1:** 5套全基因组数据集的基本情况 Characteristics of datasets selected in the studies

GEO accession	Contributor	Year	Chip	Experimental design	Probs	Source	Disease	Normal
GSE18842	Sanchez-Palencia A *et al*^[5]^	2011	HG-U133_Plus_2	Paired, tissues	54675	Homo sapiens	45	45
GSE19188	Hou J *et al*^[6]^	2010	HG-U133_Plus_2	Unpaired, tissues	54675	Homo sapiens	91	65
GSE7670	Su LJ *et al*^[7]^	2007	HG-U133A	Paired, tissues	22283	Homo sapiens	27	27
Paried: compare non-small cell lung cancer (NSCLC) to normal controls from the same patients with NSCLC; Unparied: compare NSCLC from men with NSCLC to normal controls from men without NSCLC.								

### 1.2 方法

GSEA方法通过分析2组以上的样本之间差异表达基因，对样本进行聚类以获得明显基因表达差异的样本分类。用R语言来处理数据，进行统计分析，得到数据共同改变的通路。*meta*方法对单套数据集进行*t*检验，将结果行*meta*分析，得到差异表达的基因，放入可视化综合发现注解数据库（The Database for Annotation, Visualization and Integrated Discovery, DAVID）网站得到这些基因可能所在的通路。首先通过Bioconductor^[8]^的2.10.1版本来对数据进行标准化处理。用软件包affty中的RMA算法^[9, 10]^对affymetrix平台的原始数据进行背景校正、标准化和Log2转换。然后对每一套数据每个探针的检验采用成组*t*检验，仅选取在日本基因和基因组百科全书（kyoto encyclopedia of genes and genomes, KEGG）数据库^[11]^中存在的基因进行GSEA分析，剔除变异四分位距 < 0.5的基因，如果一个基因对应几个探针，只保留变异内距（inter-quartile range, IQR）最高的探针。GSEA通过Bioconductor的category包进行，只有超过10个基因的类保留，通过*t*检验对每一个通路中的基因进行检验，通过1, 000次循环的排列组合（permutation）获得每个通路的*P*值，运用SAS 9.13软件，通过*t*检验把3套数据共同通路里的每个探针算出P值，再通过公式^[12]^ $ LS = \frac{{\sum\limits_{i = 1}^n {(-\log ({P_i}))} }}{N}$（自由度为数据集K的2倍）算出每个基因的卡方值，最后保留*P* < 0.05的基因。对这些基因通路的分析通过DAVID（http://david.abcc.ncifcrf.gov）中的KEGG库进行分析。

## 2 结果

### 2.1 GSEA分析

应用GSEA方法对3套数据集进行功能基因富集，GSE19188数据集富集出上调通路139条，下调通路40条；GSE7670数据集富集出上调通路106条，下调通路24条；GSE18842数据集富集出上调通路112条，下调通路57条。其中数据集GSE19188和数据集GSE18842通路重叠性比较高。通过3组数据中所得通路进行对比，上调中皆有的通路87条，下调中皆有的通路22条。

### 2.2 *meta*分析

运用成组*t*检验对3套数据集单独分析得出每个基因的P值后，通过软件SAS 9.13运用选择的*meta*公式进行整合分析，共筛出1, 177个基因（*P* < 0.05）。通过DAVID的KEGG库进行通路富集，这1, 177个基因中有162个基因能够在KEGG库中被筛出，主要分布在下面的19条通路中：癌症通路（pathways in cancer）、粘着斑通路（focal adhesion）、细胞骨架肌动蛋白调控通路（regulation of actin cytoskeleton）、胞吞作用通路（endocytosis）、Fc-γ-R介导吞噬作用通路（Fc gamma R-mediated phagocytosis）、胰岛素信号通路（insulin signaling pathway）、溶酶体通路（lysosome）、白细胞跨内皮迁移信号通路（leukocyte transendothelial migration）、粘着连接通路（adherens junction）、神经营养因子信号通路（neurotrophin signaling pathway）、细胞外基质受体作用通路（ECM-receptor interaction）、前列腺癌通路（prostate cancer）、长期增强作用通路（long-term potentiation）、肾细胞癌通路（renal cell carcinoma）、精氨酸和脯氨酸代谢通路（arginine and proline *meta*bolism）、致病大肠杆菌感染通路（pathogenic *Escherichia coli* infection）、神经胶质瘤通路（glioma）、膀胱癌通路（bladder cancer）、蛋白酶复合体通路（proteasome）。

### 2.3 两种方法所得结果分析

应用GSEA和*meta*两种方法得到重叠性较高的通路：粘着斑通路（图 1）和细胞骨架肌动蛋白调控通路（图 2），且该两条重要通路都属于上调通路。通过R命令语言，得到3组数据集里粘着斑通路和细胞骨架肌动蛋白调控通路各自所含基因探针号。将探针号传至DVID（http://david.abcc.ncifcrf.gov/conversion.jsp）数据库进行官方名称转换，得到3组数据里该通路所含的基因名称。GSE19188里在粘着斑通路所含差异基因152个，GSE7670含135个，GSE18842含152个；GSE19188里在细胞骨架肌动蛋白调控通路所含差异基因141个，GSE7670含118个，GSE18842含135个。通过上步*meta*运行结果可得粘着斑通路中差异有统计学意义（*P* < 0.05）的基因31个，细胞骨架肌动蛋白调控通路中差异有统计学意义（*P* < 0.05）的基因32个（表 2）。

**1:**
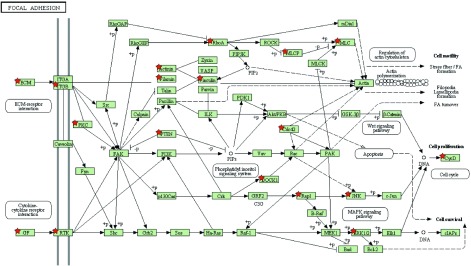
粘着斑通路示意图（图片来源于从DAVID富集出中的KEGG数据库http://www.genome.jp/dbget-bin/www_bget?map04510，^★^*P* < 0.05且在该通路中的基因） Focal adhesion pathway (The chart is from KEGG database, http://www.genome.jp/dbget-bin/www_bget?map04510, ^★^*P* < 0.05)

**2:**
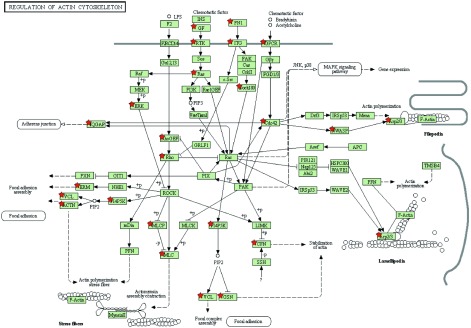
细胞骨架肌动蛋白调控通路示意图（图片来源于从DAVID富集出中的KEGG数据库http://www.genome.jp/dbget-bin/www_bget?map04810，^★^*P* < 0.05且在该通路中的基因） Regulation of actin cytoskeleton (The chart is from KEGG database, http://www.genome.jp/dbget-bin/www_bget?map04810, ^★^*P* < 0.05)

**2:** 粘着斑通路和细胞骨架肌动蛋白调控通路中*meta*分析基因分布 The gene distribution of focal adhesion and regulation of actin cytoskeleton pathways by *meta*-analysis

Gene	Focal adhesion			Regulation of actin cytoskeleton	
	Ratio	*P*		Ratio	*P*
*thbs2*	1.31	1.14×10^-14^		-	-
*COL1A1*	1.03	1.52×10^-12^		-	-
*MYL9*	0.85	1.94×10^-12^		0.85	1.94×10^-12^
*Pdgfb*	0.89	2.39×10^-10^		0.89	2.39×10^-10^
*SPDYA*	0.92	2.40×10^-10^		0.92	2.40×10^-10^
*MYL12A*	0.95	1.02×10^-9^		0.95	1.02×10^-9^
*ACTN2*	0.95	3.80×10^-7^		0.95	3.80×10^-7^
*Rap1b*	0.96	4.01×10^-6^		-	-
*LAMA4*	0.87	7.45×10^-6^		-	-
*MAPK1*	0.98	1.11×10^-5^		0.98	1.11×10^-5^
*IGF1R*	0.98	2.08×10^-5^		-	-
*PTENP1*	0.94	1.77×10^-4^		-	-
*FLNA*	0.94	2.27×10^-4^		-	-
*Mapk9*	0.96	4.52×10^-4^		-	-
*TNXB*	0.71	7.04×10^-4^		-	-
*pdgfra*	0.93	1.41×10^-3^		0.93	1.41×10^-3^
*actn4*	0.92	1.46×10^-3^		0.92	1.46×10^-3^
*Itgb5*	0.93	1.50×10^-3^		0.93	1.50×10^-3^
*ITGB6*	0.87	3.96×10^-3^		0.87	3.96×10^-3^
*Thbs1*	0.84	7.75×10^-3^		-	-
*DOCK1*	0.98	1.22×10^-2^		0.98	1.22×10^-2^
*Rhoa*	0.95	1.36×10^-2^		0.95	1.36×10^-2^
*CCND2*	0.91	1.83×10^-2^		-	-
*vcl*	0.94	2.41×10^-2^		0.94	2.41×10^-2^
*LAMA3*	0.92	2.61×10^-2^		-	-
*Prkca*	0.91	2.68×10^-2^		-	-
*fn1*	0.89	2.95×10^-2^		0.89	2.95×10^-2^
*Cdc42*	0.96	3.60×10^-2^		0.96	3.60×10^-2^
*Lama2*	0.88	3.84×10^-2^		-	-
*Col5a1*	1.01	3.89×10^-2^		-	-
*PPP1R12A*	0.93	4.33×10^-2^		0.93	4.33×10^-2^
*Arhgef1*	-	-		0.97	1.64×10^-3^
*ARHGEF12*	-	-		0.94	2.87×10^-3^
*arpc1a*	-	-		1.02	5.13×10^-6^
*ARPC4*	-	-		0.92	3.22×10^-2^
*ARPC5*	-	-		0.89	2.00×10^-2^
*CFL1*	-	-		1.01	9.90×10^-3^
*CHRM3*	-	-		0.99	1.94×10^-2^
*Gsn*	-	-		0.92	3.37×10^-8^
*IQGAP2*	-	-		0.87	3.61×10^-9^
*ITGAL*	-	-		0.83	4.28×10^-12^
*Kras*	-	-		0.94	1.70×10^-2^
*msn*	-	-		0.86	1.74×10^-9^
*Pikfyve*	-	-		0.95	3.94×10^-2^
*PIP4K2B*	-	-		0.99	1.17×10^-2^
*Scin*	-	-		0.99	2.72×10^-3^
*WASL*	-	-		0.97	1.51×10^-2^

## 3 讨论

随着基因芯片研究的广泛开展，对基因芯片数据的分析成为了基因芯片研究的重要部分。基因富集方法通过分析一组处于两种不同生物状态（如正常和癌变）的芯片数据，推断已表达的基因是否有共同的表达趋势，以此来找出与疾病关联的基因和通路^[13]^。单独对某次实验结果进行分析，且只是对单个基因进行分析，由于样本问题，可能会漏掉很多有用的信息；并且对基因芯片单套的*t*检验有一定的局限性，受到样本量的限制，导致不可信的变异估计，可产生较高的假阳性，忽略了不同样本中表达水平的差异^[14]^。本研究结合GSEA和*meta*两种方法对该3套数据进行分析，两种结果重叠对比，找出了影响NSCLC相关的重要基因和通路，最终得到重叠性较高的粘着斑通路和细胞骨架肌动蛋白调控通路以及差异性明显的重要基因。粘着斑是细胞骨架的一个重要结构，由细胞膜外的粘附素、细胞膜上的整联蛋白和细胞内的细胞骨架蛋白等相互连接集聚而成。细胞正是依靠“粘着斑”这种特殊结构将细胞嵌合在体内正确位置，从而保持细胞的正常结构并发挥其正常功能^[15, 16]^。另外，细胞与细胞间、细胞与基底膜之间的连接都与细胞骨架蛋白相连，细胞骨架功能的变化可以导致细胞形态、细胞-细胞间和细胞-基底膜粘附状态的改变；同时，细胞-细胞间和细胞-细胞基底膜粘附功能状态的变化通过细胞信号转导机制导致骨架蛋白的重新排列，最终引起内皮通透性的改变。内皮细胞形态变化和收缩性的改变主要受骨架蛋白如肌动蛋白和肌球蛋白的影响。内皮细胞收缩性的改变被认为是不同的信号和机制导致通透性变化的最后共同通路。目前已有多项研究^[17]^显示，蛋白激酶C（protein kinase C, PKC）与激动蛋白（actin）的结合可以激活PKC，PKC的激活促进粘着斑的形成，其机制包括增加粘着斑激酶（FAK）的活性、促进整合素的碱性化以及调节其它相关蛋白的功能等。其次，在我们筛选出的粘着斑通路31个差异基因和细胞骨架肌动蛋白调控通路32个差异基因中，两条通路共有且*P* < 0.01的基因有*ACTN2*、*ITGB6*、*Itgb5*、*MAPK1*、*MYL12A*、*MYL9*、*Pdgfb*、*SPDYA*、*actn4*、*pdgfra*等，预测这10个基因可能与NSCLC的发生发展有密切联系。通过查找文献发现MAPK1^[18]^、Pdgfb^[19]^和pdgfra^[20]^与NSCLC发生发展有密切关系，余下的7个基因没有找到其与NSCLC的相关报道。我们还通过KEGG通路数据库、*P* < 0.05以及文献报道找出粘着斑通路中起关键作用的基因有*ECM*、*ITGB*、*PKC*、*PTEN*、*ERK1/2*、*JNK*、*GF*、*RTK*等，细胞骨架肌动蛋白调控通路中起关键作用的基因有*ERK*、*GF*、*RTK*、*Ras*、*Rac*等，这些基因均与肿瘤的发生发展具有密切的关系，并且其中部分基因与NSCLC的发生发展具有密切的关系，后续将通过实验来验证其与NSCLC发生发展之间的具体联系。

本研究对GEO基因芯片数据库目前能找到且已有文献支持的人类肺组织标本全基因组表达芯片进行了研究，已筛选出可能与NSCLC的发生发展具有密切关系的基因和通路，但是基因和通路的数量过多，这可能与数据集数量以及所包含的标本量有关，并且本研究所得结果仍然只是运用生物信息学对NSCLC发生发展的重要基因和通路的预测，后续研究小组将对这些差异明显的基因进行生物功能学的验证，以求从根本上发现与NSCLC发生发展相关的重要基因和通路，为NSCLC发病遗传机制及靶向治疗的研究奠定科学基础。
